# Lactic Acid Fermentation, Urea and Lime Addition: Promising Faecal Sludge Sanitizing Methods for Emergency Sanitation

**DOI:** 10.3390/ijerph121113871

**Published:** 2015-10-29

**Authors:** Catherine Anderson, Dennis Hanjalika Malambo, Maria Eliette Gonzalez Perez, Happiness Ngwanamoseka Nobela, Lobke de Pooter, Jan Spit, Christine Maria Hooijmans, Jack van de Vossenberg, Wilson Greya, Bernard Thole, Jules B. van Lier, Damir Brdjanovic

**Affiliations:** 1 WASTE advisers on urbane environment and development, Lange Houtstraat, 26, 2511 CW, The Hague, The Netherlands; E-Mails: catherine.anderson85@gmail.com (C.A.); jspit@waste.nl (J.S.); 2 UNESCO-IHE, Westvest 7, 2611 AX Delft, The Netherlands; E-Mails: dmalambo@lwsc.com.zm (D.H.M.); eliettegonzalez710@gmail.com (M.E.G.P.); hnobela@yahoo.com (H.N.N.); J.vandeVossenberg@unesco-ihe.org (J.V.); J.B.vanLier@tudelft.nl (J.B.L.); d.brdjanovic@unesco-ihe.org (D.B.); 3 Delft University of Technology (TUDelft), Stevinweg 1, 2628 CN Delft, The Netherlands; E-Mail: lobke.depooter@gmail.com; 4 The University of Malawi—The Polytechnic, Ginney Corner, Blantyre 3, Malawi; E-Mails: wilsongreya@gmail.com (W.G.); bthole@poly.ac.mw (B.T.)

**Keywords:** ammonia, emergency sanitation, *Escherichia coli*, excreta, faecal sludge, lactic acid, lime, urea

## Abstract

In this research, three faecal sludge sanitizing methods—lactic acid fermentation, urea treatment and lime treatment—were studied for application in emergency situations. These methods were investigated by undertaking small scale field trials with pit latrine sludge in Blantyre, Malawi. Hydrated lime was able to reduce the *E. coli* count in the sludge to below the detectable limit within 1 h applying a pH > 11 (using a dosage from 7% to 17% *w*/*w*, depending faecal sludge alkalinity), urea treatment required about 4 days using 2.5% wet weight urea addition, and lactic acid fermentation needed approximately 1 week after being dosed with 10% wet weight molasses (2 g (glucose/fructose)/kg) and 10% wet weight pre-culture (99.8% pasteurised whole milk and 0.02% fermented milk drink containing *Lactobacillus casei* Shirota). Based on Malawian prices, the cost of sanitizing 1 m^3^ of faecal sludge was estimated to be €32 for lactic acid fermentation, €20 for urea treatment and €12 for hydrated lime treatment.

## 1. Introduction

The provision of clean water and sanitation, which are essential services for safeguarding public health, are often disrupted in emergency situations. In comparison to drinking water, sanitation is often assigned a lower priority within an emergency context. This in addition to many other factors has led to the provision of unsuitable on-site sanitation provision, during emergency response, particularly in urban areas [1]. Outbreaks of diarrhoeal diseases including dysentery and cholera are common in emergencies [2]. Faecal-oral (orofaecal) route of transmission of a disease may account for more than 40% of mortalities in the acute phase of an emergency, with greater than 80% of mortality of children under 2 years of age [3]. Containment and treatment of faecal matter is a vital barrier against the spreading of diarrhoeal diseases in particular during emergencies, when the affected population is more vulnerable [4].

The research conducted has been done in response to a call to investigate faecal sludge treatment options that could be rapidly deployed upon the event of an emergency and are effective under challenging physical site conditions *e.g.*, densely populated urban area, unstable soil, high water table and flood-prone area.

Three faecal sludge treatment methods were investigated, namely: application of lactic acid fermentation, urea and lime. All three treatment processes require readily available material: molasses (a common livestock feed), urea (a common fertilizer) and hydrated lime (a common building material); therefore have the potential for rapid deployment and implementation upon the event of an emergency.

The objectives of this study were to determine the sanitizing effect of lactic acid fermentation, urea and lime faecal sludge treatment methods on the population of *E. coli* as the indicator organism and to assess the applicability of these treatment methods to emergency situations through undertaking field trials in Malawi.

Lactic acid bacteria (LAB) pose the ability to convert carbohydrates to lactic acid and the genera *Lactobacillus*, *Streptococcus* and *Leuconostoc* are readily used for sanitation within the food and fermentation industries [5]. The antimicrobial action of the metabolite lactic acid is partially attributed to its ability to penetrate the cytoplasmic membrane of microorganisms in the associated form, resulting in a reduced intracellular pH and disruption of the trans-membrane proton motive force of the lipopolysaccharides molecules of the outer membrane of the (pathogenic) organism [6]. Additionally, lactic acid reduces the bulk pH of the surrounding medium, which influences the activity of exo-enzymes and membrane-bound enzymes. A study by Zhu *et al.* [7] reported that whilst the survival of bacteria was diminished at pH less than 3.5, killing of bacteria required a pH of less than 2.5. The fact that lactic acid reduce the bulk pH to approximately pH 4, indicates that rather than reducing the extracellular pH, the key antimicrobial property of lactic acid is its ability to reduce the intracellular pH of bacteria.

Whilst numerous studies have been conducted to investigate the sanitizing potential of lactic acid within the food industry [8], studies specifically focusing on the sanitizing effect of lactic acid on pathogens present in faecal sludge are limited. A study conducted by Ligocka and Paluszak [9], demonstated that *Salmonella* spp. and *E. coli* in sewage sludge were inhibited under both anaerobic and aerobic conditions with lactic acid bacteria of the genus *Lactobacillus*. Under aerobic conditions the metabolites lactic acid and hydrogen peroxide were of the most influential for limiting pathogen growth whereas bacteriocins displayed the most antagonistic effect on selected pathogenic bacteria under anaerobic conditions. Soewondo *et al.* [10] conducted laboratory experiments treating faeces sourced from a urine diverting dry toilet (UDDT) by lacto-fermentation using a non-specified microbial culture (EM4) as the inoculum and 0%, 5%, 10% glucose as the co-substrate. Total coliforms were used as the sanitation indicator and the initial count was of the magnitude log 8 Total Coliform CFU/mL. After 21 days, the addition of EM4 with 0%, 5% and 10% *w*/*w* glucose resulted in a log reduction in total coliforms of Log 4, Log 5.5 and Log 7.5, respectively.

Urea treatment is based on the sanitizing effect of increased pH in combination with cell alkalisation by uncharged ammonia (NH_3_) [11]. Ammonia efficiently inactivates bacteria at pH 9–9.5 by entering the cell membrane, increasing the internal ammonia concentration and causing the bacterial cell to sacrifice protons to maintain its optimum cellular pH until eventually resulting in cell death [12]. The enzyme urease is required for the conversion of urea into ammonia [13]. Urease is an enzyme produced by bacteria living in soil, aquatic environments and in the human intestinal tract, hence urease is also present in faeces [14]. The amount of uncharged ammonia depends on urea added and conversion rate, which is guided by temperature and pH and the medium. The amount of unionized ammonia is also dependent upon the equilibrium with ammonia gas, therefore head space and ventilation are also important [15].

Previous urea treatment studies have indicated a reduction in numbers of organisms, including non-spore forming bacteria, viruses and parasites through urea additions to manure and faecal sludge [15,16]. In a study by Vinnerås [17], urea (3% N-NH_3_) was added to faecal matter (10% dry matter slurry). The pH increased to 9.2 within 1 h, *Salmonella* spp. and faecal coliforms were not detected after 5 days, *Enterococcus* spp. were not detected after 20 days and viruses as well as viable *Ascaris* eggs were not detected after 50 days of treatment.

Lime treatment is readily applied to treat wastewater treatment sludges and involves the application of a hydrated (slack) lime (Ca(OH)_2_) to create an alkaline environment which is hostile to biological activity. At pH levels greater than 12, the cell membranes of harmful pathogens are destroyed [18]. The high pH also leads to high fractions of non-ionised ammonium which, as detailed in the previous urea treatment section, will act as a biocide and contribute to pathogen removal if contained within the reactor [19].

Previous studies describe the effectiveness of lime in reducing microbiological hazards in wastewater [20]. The work of Bina *et al.* [18] investigated the removal of faecal coliforms, *Salmonella* and helminth eggs using lime treatment at pH 11 and pH 12. The sanitation requirements for faecal coliform (<1000 MPN/g DS) and *Salmonella* (<3 MPN/4 g) were achieved at pH 12. Although the sanitation requirement for faecal coliforms was achieved initially within 24 h at pH 11, re-growth of bacteria was observed after 72 h of storage. It was concluded that lime treatment was ineffective at reducing helminth eggs at both pH 11 and pH 12.

A recent case study investigating lime stabilisation upon faecal sludge was conducted in the Philippines and reported in Strande *et al.* [21]. In this study, disinfection was achieved after 30 min at pH 12, after 60 min at pH 11.5 and after 120 min at pH 11. Quality of lime, sludge characteristics and extent of mixing were the key factors affecting the process conditions, rate of pH increase and final pH.

## 2. Experimental setup

### 2.1. Origin of Faecal Sludge

The field research was conducted in Blantyre, Malawi at the Sochi sewage treatment plant. The faecal sludge used in the experiments originated from market and household pit latrines; the characteristics of which are detailed in Table 1. The Bangwe market facility, from where sludge batches 2–5 were taken, consists of two pit latrines with approximately 50–80 users per day which is a similar user density to the initial acute phase of emergency situations as defined by the sphere project which states a usage of 20–50 people per latrine [22].

**Table 1:** Characteristics of initial faecal sludge used in the field experiments.

Property	Sludge 1 LA, U	Sludge 2 L	Sludge 3 L	Sludge 4 L	Sludge 5 U	Literature Values *
Sludge Source	Household Latrine	Market Latrine	Market Latrine	Market Latrine	Market Latrine	NA
**Sludge Age**	7 years	2 weeks	1 month	2 weeks	2 weeks	NA
**pH**	7.6	6.7	6.0	6.7	6.9	6.55–9.35
**Temperature (°C)**	21	25	23	27	15	NA
**Total Solids (%)**	5.6	10.7	11.7	14.9	8.8	3.5–5.25
**Volatile Solids (as % of TS)**	55	68	58	66	73	65
**COD (mg/L)**	NA	95,000 ± 22,500	120,800 ± 10,400	103,000 ± 9600	301,000 ± 28,000	20,000–50,000
**Ammonia-Nitrogen (mg N/L)**	NA	NA	NA	1200 ± 300	1050 ± 400	NA
***E. coli* (log CFU/mL)**	4.5	4.5	4.3	4.8	5.8	NA
**Total Coliforms (log CFU/mL)**	4.8	4.5	4.6	5.0	6.0	3.0

* All literature sources presented in Table 2 and Table 3 are from in Strande *et al.* [21]; LA, Lactic Acid Treatment Experiments; U, Urea Treatment Experiments; L, Lime Treatment Experiments.

The faecal sludge was extracted from the pit latrines the day prior to field testing. The desludging method employed consisted of fishing to remove solid matter (*e.g.*, clothes, stones, menstrual rags), followed by high pressure fluidization and extraction via a vacuum suction pump. Depending on the faecal sludge shear properties, up to 0.5 L/L of water were added to sludge during the fluidization process. The faecal sludge was transported to the field test site in an 800 L vessel mounted on a light truck. At the field testing site, a reverse suction pump was used to fill each individual 50 L plastic drums with approximately 25–35 kg faecal sludge. Prior to the commencement of the three bio-chemical treatment processes, the weight of faecal sludge in each drum was recorded and samples (100–200 g) were taken from each of the treatment drums for the physical and microbial properties to be analysed.

### 2.2. Faecal Sludge Characterization

The pH and temperature were measured via potentiometric methods (Standard method (SM) 4500-H+ in [23]) using a SENSION + PH1 Advanced Field Kit. The total solids and volatile solids were measured using Standard Method procedures for the examination of water and wastewater SM-2540D and SM-2540E respectively [23]. The Chemical Oxygen Demand (COD) was measured using the US EPA Reactor Digestion Method involving a cell test (Hach Lange High Range: 0–1500 mg COD/L) Tube test) via a reaction with sulphuric acid and potassium dichromate solution, heating to 150 °C with a thermostat (DRB200) and measurement reading using a colorimeter (DR 980). The pit latrine sludge was firstly diluted 100–1000-fold to enable the COD measurement to be within the readable range. The Total Ammonia Nitrogen (TAN) concentration was measured using indophenol blue method with the following cell tests: vario cell test 1.0–50.0 mgN/L and Hach LR TNT cell test 0.00–2.50 mgN/L and measurement reading using a colorimeter (DR 980, Düsseldorf, Germany). Because of the relative high TAN and solids concentration in the pit latrine sludge, samples were firstly diluted and filtered before analysis. Each set of COD and TAN measurements were also accompanied by a blank to calibrate the colorimeter.

The indicator organism *E. coli* was used to assess the sanitation efficiency of the lactic acid, urea and lime treatment processes. Total coliforms were also measured during the urea and lime treatment experiments. For the microbial analysis serial dilutions of the sample were undertaken in physiological saline solution. *E. coli* and total coliform count were determined via the spread plate method with Chromocult Coliform Agar, following incubation at 37 °C for 24 h (Standard Method 9020 [23]). The treatment time was defined as the time required for the *E. coli* count to reduce below the detectable limit.

For the lactic acid experiments, *Lactobacillus casei* shirota was cultured using a pour plate technique on MRS agar and incubated at 37 °C for 72 h (Standard Method 9215: [23]). The lactic acid concentration was analysed reflectometrically using Reflectoquant^®^ lactic acid test strips (Merck Millipore International, Darmstadt, Germany) and measured using a reflectometer (RQflex 10 Meter, Darmstadt, Germany).

### 2.3. Lactic Acid Fermentation Experimental Set Up

For the lactic acid experiments, a pre-culture of lactic acid bacteria was first created through mixing pasteurized whole milk with a fermented milk drink containing *Lactobacillus casei* Shirota in the ratio of 99.8% *w*/*w* and 0.02% *w*/*w*, respectively. This pre-culture was inoculated for 65 h at room temperature in order for the lactic acid bacteria to exponentially grow to levels of approximately log 7.7 CFU/mL. For the sugar additive, cane molasses was sourced from a local supplier in Blantyre and could be characterized by a total solids content of 87% and a total monosaccharides (glucose and fructose) concentration of 20,000 mg/L

The lactic acid experiment was conducted in triplicate with a single control drum using sludge 1 (refer Table 1). Based on faecal sludge wet weight, 10% *w*/*w* pre-culture and 10% *w*/*w* molasses (equivalent to 2 g simple sugars ((glucose, fructose)/kg wet sludge) were added to the three treatment drums. The four drums (control and three treatment drums) were intensely mixed using a power mixer for three minutes. All samples (100–200 g) were collected from the top of the treatment reactor and analyzed for pH, lactic acid concentration and *E. coli* count.

### 2.4. Urea/Ammonia Treatment Experimental Set Up

The urea treatment experiment was conducted twice once with household sludge (sludge 1, Table 1) and again with market sludge (sludge 5, Table 1). The experimental set up consisted of two plastic drum reactors: one control and one treatment reactor. Urea prills sourced from a local agricultural dealer in Blantyre, Malawi were added to the treatment reactors at a dosage of 2.5% *w*/*w* urea based on sludge wet weight. In the first experiment with household sludge (sludge 1), the treatment reactor and control reactor were hermetically sealed with an aluminium ring subsequent to urea addition to avoid ammonia loss and then manually mixed for 3 min. Analysis of the first experiment revealed that urea hydrolysis takes approximately two days, therefore the risk of ammonia loss during the first 30 min after urea addition was deemed low and in the second experiment, which utilized market sludge (sludge 5), the drum reactors were mixed with a power mixer for three minutes before being hermetically sealed with an aluminium ring to avoid ammonia loss throughout the experiment. All samples (100–200 g) were taken from the tap at the base of the drum to guarantee no ammonia extrusion and analysed for temperature, pH, ammonia concentration, *E. coli* count and total coliform count. Sampling from the base of the drum was deemed representative based on an initial experiment (data not shown) which concluded that there was no significant difference found in samples taken from the top or bottom of the drum. The similar pH and ammonia concentration in the top and bottom layer indicate that initial mixing results in a relatively homogeneous NH_3_ distribution after three days. This is in line with the limited NH_3_ transport found for sludge with a total solid concentration of 15% (5 cm in 3 days) [24].

### 2.5. Lime Treatment Experimental Set Up

The hydrated lime treatment experiments were conducted on three separate occasions with three different sludge batches (sludges 2–4, Table 1). The experiments were based on pH control and consisted of five plastic drum reactors. Initially a sludge sample (200 g) was taken from each of the drums and the buffer capacity of the sludge batch determined through incrementally adding lime to the sample whilst monitoring the pH until the pH reached 12.5. From the lime dosage-pH relationship determined through the small batch experiment, the proposed lime dosage required to achieve the target pH conditions in each of the drums was estimated. For each of the experimental runs, a single drum reactor acted as a control and hydrated lime sourced from a local hardware store was added to the four remaining reactors to achieve a different fixed target pH condition within the pH range between 9 and 12.3 in each drum (*i.e.*, 9 or 10 or 11.5, *etc.*). In the four treatment reactors the lime was mixed with the faecal sludge for 10 min using an electric power mixer. All samples (100–200 g) were taken from the top of each of the five drums and analyzed for temperature, pH, *E. coli* count and total coliform count.

## 3. Results and Discussion

### 3.1. Faecal Sludge Characteristics

The physical and microbial properties measured for the faecal sludge used in this study are presented in Table 1. Characteristics of faecal sludge produced during an emergency situation have not been recorded and will differ depending on several factors such as people’s diets, temperature, the sanitation system employed, desludging frequency, anal cleansing method *etc.* When compared to literature values obtained for faecal sludge reported in Strande *et al.* [21], the sludge used in the field trials was characterised with higher solids contents, COD concentrations and coliform counts.

The difference in total solids content between sludge 2 to 5, which were extracted from the same pit latrine at different times, illustrates the seasonal variance in sludge characteristics and the impact of other factors such as ground water intrusion and water addition during pit emptying. Comparing the properties of sludge 1 with sludge batches 2–5 shows the differences between household and market pit latrine sludge and the impact of factors such as storage duration upon sludge characteristics. Whilst the degree of stabilization (volatile solids;VS/total solids;TS ratio) was higher for household sludge compared to market sludge, the *E. coli* and *total coliforms* count were approximately of the same order of magnitude for both sludge types. For the field experiments, the tests with lactic acid and urea were carried out using sludge 1—the first faecal sludge batch that was collected. Market sludge was procured for the subsequent experiments with the three hydrated lime tests undertaken, using three separate sludge batches, namely: sludges 2, 3 and 4. Five months later, an additional urea test was undertaken using market sludge from the same market pit latrine-sludge batch 5.

### 3.2. Lactic Acid Fermentation Experimental Results

A summary of the lactic acid fermentation trials with sludge 1 is given in Table 2. Over the course of the experiment, the lactic acid concentration in the treatment reactors increased, most likely due to the lactic acid bacteria metabolizing the sugars in the added molasses, producing lactic acid. The lactic acid concentration did not decrease during the experiment and the concentration appears to reach a plateau at 47 g/L after 1 week (~168 h). The increase in lactic acid concentration coincides with a decrease in the pH level over the course of the experiment with the pH stabilizing at approximately pH 4.2 after 1 week. This is consistent with the results reported by Soewondo *et al.* [10] who recorded approximately pH 4.5 after 7 days of lacto-fermentation treatment of fecal matter by EM4 and 5% glucose.

Comparing the control and treatment reactors it can be seen that there is an initial increase in *E. coli* count for the treatment reactors in 0–96 h which might be attributed to the addition of a carbon sources (molasses) to the treatment reactors. The suppression of the *E. coli* count to below detectable numbers was noted in all three of the treatment reactors after 168 h. The decline in the viable colony count of *E. coli* to below the detection limit between 96 h and 168 h, coincides with the increase in lactic acid concentration and possibly other metabolites produced by the lactic acid bacteria. The *E. coli* concentration reduced below detectable limit of log 2 CFU/mL within 168 h when the lactic acid concentration was above 35 g/L and the pH was in the range of pH 4.2. The >2.5 log reduction within 168 h observed in this study is of a similar magnitude to the log reduction in pathogenic bacteria recorded by Soewondo *et al.*, ([10]) for lactic acid fermentation treatment of fecal matter with EM4 and 5% glucose.

Comparing literature, initial laboratory experimental results (data not shown) and results of the field trial, two key factors that were observed to affect the sanitizing time and the extent of lactic acid fermentation faecal sludge treatment are faecal sludge alkalinity and substrate composition (e.g., glucose vs molasses). The alkalinity of faecal sludge impacts the rate of change in pH which in turn determines the proportion of lactic acid present in the deionized (and biocidal) form as well as subsequent sanitization affects. Substrate composition and in particular the concentration of simple sugars in the substrate impact the growth rate of lactic acid bacteria as well as the quantity and rate of lactic acid production, which in turn impacts the rate and extent of pathogen reduction.

**Table 2:** Process monitoring of lactic acid fermentation process in the field with sludge 1 (cf. Table 1).

Treatment Time (h)	Field Trials (Faecal sludge volume: 25 L and Temperature 20-25 °C) LATR : 10% Molasses (2 g/L Glucose/Fructose), 10% LAB Preculture (Wet Sludge Weight)					
	pH		Lactic Acid Concentration (mg/L)		*E. coli* (log CFU/mL)	
	CR	LAR	CR	LAR	CR	LAR
**0**	7.6	7.8	62	82 ± 2	4.59 ± 0.01	4.35 ± 0.35
**48**	7.4	5.6	72	21 ± 1 × 10^4^	4.64 ± 0.03	6.17 ± 0.04
**96**	6.9	4.6	75	35 ± 4 × 10^4^	4.38 ± 0.09	4.87 ± 1.01
**168**	6.9	4.2	77	47 ± 1 × 10^4^	4.23 ± 0.05	not detected *
**216**	6.9	4.2	68	47 ± 2 × 10^4^	3.00 ± 0.09	not detected *

CR = control reactor, LAR = Lactic Acid treatment Reactor (average and standard deviation calculated from the variance in the three treatment reactors). * Detection limit was log_10_ 2 CFU/mL.

About 94% of the cost of lactic acid treatment, *i.e.*, €31/m^3^, is associated with the expensive pre-culture (99.8% *w*/*w* pasteurized whole milk 0.02% *w*/*w* fermented milk drink containing *Lactobacillus casei* Shirota). The pre-culture used in the field trial had a lactic acid concentration of 16 g/L, a pH level of 3.8 and a lactic acid bacteria count of approximately log 7.71 CFU/mL. Field trial results indicated that the lactic acid concentration of the treated sludge (47 g/L) was greater than that of the original inoculant (16 g/L), therefore the expensive pre-culture potentially could be replaced by the treated faecal sludge for subsequent inoculations reducing the costs to approximately € 2/m^3^ for subsequent treatment processes. Further testing is required to prove this hypothesis.

### 3.3. Urea/Ammonia Treatment Experimental Results

For the experiment utilizing household sludge (sludge 1), the *E. coli* count was reduced to below detectable limits within 96 h (4 days) when the ammonia nitrogen concentration was between 4500–5800 mgN/L and the pH was approximately 9 (Table 3). The total coliform count was reduced to below detectable limit within 168 h (7 days) when the ammonia nitrogen concentration was between 5800 and 7700 mgN/L and pH approximately 9.2. Similar results were observed for the subsequent experiment utilizing market sludge (sludge 5, Table 3) with E. coli and total coliforms not being detected after 96 h (4 days) and 168 h (7 days) respectively at pH 9.4. A similar deactivation period of 120 h (5 days) for bacterial pathogens and pH 9.2 conditions were reported in the study by Vinnerås [17] utilizing urea (3% N-NH_3_).

The addition of 2.5% *w*/*w* urea (based on wet weight) resulted in a pH rise of approximately 1.3 over the first 48 h and eventual stabilization at pH 9.2 and 9.4, respectively, for household and market sludge within 168 h. The ammonia concentration increased throughout the entire 168 h experiment utilizing household sludge (sludge 1), most likely due to the intrinsic urease within the faecal sludge catalysing urea hydrolysis to form ammonia as well as induce the favourable pH conditions (for the equilibrium with ammonium). The ammonia-nitrogen concentration rose to 4500 mgN/L within 48 h and 7700 mgN/L within 168 h of urea treatment with sludge 1. The significant rise in ammonia concentration suggests that the process was not urease limited, but that sufficient urease was present in the faecal sludge to hydrolyse 2.5% *w*/*w* urea in the case of sludge 1.

**Table 3:** Process monitoring of the urea/ammonia treatment process in the field with sludge 1 and sludge 5 (cf. Table 1).

Treatment Time (h)	Field Trials (Faecal Sludge Volume: 25 L and Temperature 20–25 °C) UTR: 2.5% *w*/*w* Urea (Based on Sludge Wet Weight)							
	**pH**				**Ammonia Nitrogen(mgN/L) ^a^**			
	**Household Sludge 1**		**Market Sludge 5**		**Household Sludge 1**		**Market Sludge 5**	
	**CR**	**UR**	**CR**	**UR**	**CR**	**UR**	**CR**	**UR**
0	7.4	7.7	6.9	7.0	67	77	NA	NA
48	7.0	9.0	6.7	8.3	19	4.5 × 10^3^	NA	NA
72	NA	NA	6.8	8.7	NA	NA	NA	NA
96	7.1	9.1	6.8	9.5	16	5.8 × 10^3^	NA	NA
168	7.3	9.2	7.1	9.4	17	7.7 × 10^3^	NA	NA
	***E. coli* (log CFU/mL) ^b^**				**Total Faecal Coliforms (log CFU/mL) ^b^**			
	Household sludge 1		Market sludge 5		Household sludge 1		Market sludge 5	
	CR	UR	CR	UR	CR	UR	CR	UR
0	3.95 ± 0.16	3.95 ± 0.16	5.85 ± 0.10	5.70 ± 0.08	4.00 ± 0.78	4.00 ± 0.78	6.00 ± 0.30	5.78 ± 0.07
48	NA	NA	5.70 ± 0.26	3.00 ± 0.30	NA	NA	6.30 ± 0.18	3.95 ± 0.12
72	NA	NA	5.90 ± 0.05	3.48 ± 0.12	NA	NA	5.95 ± 0.05	3.78 ± 0.18
96	3.00 ± 0.30	not detected ^c^	5.60 ± 0.18	not detected ^e^	2.95 ± 0.33	2.90 ± 0.05	6.00 ± 0.30	3.48 ± 0.12
168	2.85 ± 0.23	not detected ^d^	5.90 ± 0.05	not detected ^e^	2.30 ± 0.54	not detected ^d^	6.30 ± 0.18	not detected ^e^

CR = control reactor, UR = urea treatment reactor (2.5% *w*/*w* urea), NA = not available, ^a^ The ammonia-nitrogen was calculated multiplying the Total Ammonia Nitrogen b (TAN) by the fraction of TAN present as free non ionized ammonia given by the Henderson-Hasselbalch equation: $f_{{NH}_{3}}=\frac{1}{\left(10^{pKa-pH}+1\right)}$, *where t*he balance between ammonium ions and dissolved ammonia is given by the pK_a_. $pK_{a}=\frac{2729.92}{T}+0.0901821$, *T measured in Kelvin* [25]; ^b^ The values given represent the average ± standard deviation within the sample duplicates, except where the plate count was below the viable count (<25/plate) and the error was calculated using the method described in [26]; ^c^ Detection limit log_10_ 2 CFU/mL; ^d^ detection limit log_10_ 1 CFU/mL; ^e^ detection limit log_10_ 3 CFU/mL.

An additional urea treatment experiment was conducted with market sludge (data not shown) using sludge extracted from the same Bangwe market latrine as sludge 5. The experiment recorded the increase in ammonia-nitrogen concentration over a period of 72 h after 2% *w*/*w* (wet basis) urea addition to a treatment reactor. The initial ammonia-nitrogen concentration was approximately 25 mgN/L (pH 7.5, 20 °C) and increased to 1200 mgN/L (pH 8.9, 20 °C), 3000 mgN/L (pH 9.2, 20 °C) and 4500 mgN/L (pH 9.3, 20 °C) after 24, 48 and 72 h, respectively. The ammonia concentration of the control reactors remained in the range of 20–70 mgN/L over the 72 h period. The rate of urea hydrolysis and subsequent ammonia-nitrogen concentration increase was comparable with the initial household sludge experiment using sludge 1 and the subsequent experiment using market sludge from Bangwe market. One can speculate that for both household and market sludge cases urea hydrolysis was not rate limiting and the enzyme urease was sufficiently abundant for hydrolysis using 2%–2.5% *w*/*w* urea.

### 3.4. Hydrated Lime Treatment Experimental Results

Three separate hydrated lime experiments were conducted utilising sludge batches 2, 3 and 4, the characteristics of which are detailed in Table 1. The results of these three experiments are collated based on target pH and are summarized in Table 4.

Comparing the individual hydrated lime experiments (sludges 2–4), it was noted that the sludge characteristics impacted the amount of lime required to be added to the faecal sludge in order to achieve the specific target pH in each treatment reactor (see the range of % *w*/*w* ds for each pH in Table 4). This is most likely due to the variance in alkalinity between the separate pit latrine sludge batches. The microbial reduction observed at a specific target pH, however, was comparable across all experiments utilising the different sludge batches.

**Table 4:** Process monitoring of hydrated lime treatment in the field with sludges 3–6 (cf. Table 1).

	Field Trials (Faecal Sludge Volume: 25 L and Temperature 20–25 °C) Lime Dosage for R1–5 Based on Sludge Dry Weight (Lime Dose % *w*/*w* DS)					
Treatment Time (h)	CR pH 6–7(0%)	LR1 pH 9 (3%–9% *w*/*w* DS)	LR2 pH 10 (5%–12% *w*/*w* DS)	LR3 pH 11 (7%–17% *w*/*w* DS)	LR4 pH 11.5 (9%–19% *w*/*w* DS)	LR5 pH 12 (10%–24% *w*/*w* DS)
	***E. coli* (log CFU/mL) ^a^**					
**0**	4.60 ± 0.10	4.70 ± 0.26	4.78 ± 0.12	4.20 ± 0.16	4.20 ± 0.12	4.60 ± 0.24
**1**	4.48 ± 0.22	4.48 ± 0.12	3.30 ± 0.18	not detected ^b^	not detected ^b^	not detected ^b^
**2**	4.60 ± 0.24	4.48 ± 0.12	2.70 ± 0.15	not detected ^b^	not detected ^b^	not detected ^b^
**5**	4.48 ± 0.30	4.08 ± 0.03	not detected ^b^	not detected ^b^	not detected ^b^	not detected ^b^
	**Total Coliforms (log CFU/mL) ^a^**					
**0**	4.78 ± 0.22	4.95 ± 0.22	4.98 ± 0.02	4.52 ± 0.04	4.30 ± 0.40	4.95 ± 0.22
**1**	4.70 ± 0.26	4.78 ± 0.18	3.78 ± 0.07	3.70 ± 0.15	3.30 ± 0.40	not detected ^b^
**2**	4.85 ± 0.27	4.60 ± 0.24	2.85 ± 0.15	3.85 ± 0.27	not detected ^b^	not detected ^b^
**5**	4.60 ± 0.24	4.18 ± 0.17	2.48 ± 0.12	3.40 ± 0.12	not detected ^b^	not detected ^b^

CR = control reactor, LR = Lime Treatment Reactor, ^a^ The values given represent the average ± standard deviation between the three experiments for the same target pH treatment reactor except where the plate count was below the viable count (<25/plate) and the error was calculated using the method described in [26]; ^b^ Detection limit log_10_ 2 CFU/mL.

The *E. coli* count was reduced to below the detectable after 5 h at pH 10 and after 1 h at pH conditions above pH 11. The total coliform count was reduced to below the detectable levels after 2 h at pH 11.5 and after 1 h at pH 12. These microbial reduction times are similar to the disinfection times of 1 h at pH 11.5 conditions reported in Strande *et al.* [21].

### 3.5. Treatment Comparison and Applicability to Emergencies

All three faecal sludge treatment methods investigated were able to satisfy the top four criteria for emergency sanitation:

(1) Safety: they can be conducted safely and adhere to the safety, health and environmental norms and standards during operation and maintenance;
(2) Sanitation: they are able to reduce *E. coli* to below detectable limit;
(3) Robustness: they can treat both liquid and solid sludge. All three technologies could be undertaken in either an above ground tank or portable bladder and therefore could be effective under challenging physical conditions such as unstable soils, high water tables and flood-prone areas;
(4) Deployment: they are low-tech and require readily available material, and therefore have the potential for rapid deployment upon the event of an emergency.

Whilst each of the treatment processes meet the key emergency sanitation criteria, their respective suitability depends on the requirements for a particular emergency situation. All three processes are relatively simple, however the biological nature of lactic acid fermentation as well as the enzyme aspect of the urea treatment make the efficiency of these two processes more sensitive to environmental conditions (e.g., temperature) and hence less robust compared to the hydrated lime treatment. Hydrated lime and lactic acid treatment processes can be undertaken in aerobic conditions and hence can take place in lined pits or an open tank, making them more versatile options for an emergency setting compared to urea, which requires a sealed vessel with minimum headspace. Furthermore, lime is the most effective treatment in terms of time, sanitizing sludge within an hour compared to urea and lactic acid treatments which require approximately four and seven days respectively.

The sanitised sludge produced from each of these three processes is very different in nature: lactic acid fermentation produces an acidic sludge that has a high content of lactic acid bacteria and hence has the potential to be used directly as an inoculant for subsequent treatment batches. The acidic sludge would require neutralisation in addition to stabilisation prior to being discharged safely into the environment. The sludge produced by the urea treatment is slightly alkaline (pH 9) and has a very high nitrogen content, therefore has potential for agricultural use. The urea-treated sludge is not stabilised and has been exposed to anaerobic conditions, therefore it is an odorous material. Hydrated lime produces a highly alkaline sludge which is not odorous, but has limited reuse potential aside from acidic soil conditioning. Lime-treated sludge would require neutralisation in addition to stabilisation prior to being discharged safely into the environment.

The chemical costs associated with each of the three sanitization processes were estimated using Malawian market prices due to the location of the field trials. Lactic acid fermentation was found to have the highest cost for the initial batch €31.2/m^3^ sanitised sludge, however the lowest cost for every subsequent batch of €2.2/m^3^ due to the potential re-use of the treated sludge. The chemical cost of 2.5% *w*/*w* urea treatment was estimated to be €20/m^3^ sanitised sludge, however it must be noted that urea is a subsided fertilizer in Malawi. The cost of lime treatment is heavily dependent upon the alkalinity of the raw faecal material and the estimated cost of sanitizing 1m^3^ of faecal sludge was €12 based on the three field trials.

Table 5 displays the results of multi-criteria analysis that was undertaken to evaluate the three different potential emergency sanitation treatment options based on their applicability to an emergency situation. Each of the emergency sanitation criterion was assigned a ranking (from 1: unimportant to 5: critical) generated in consultation with emergency response personal (Red Cross, IFRC, October, 2013). Each of the faecal sludge treatment options were then qualitatively assessed against each criterion and assigned an alternatives ranking (1 = very poor to 5 = very good). The overall score for the three sanitation technologies resulted from the summation of the product of the criterion weight and alternatives ranking. Overall this assessment revealed that each of the three treatment technologies offer benefits in an emergency situation. The qualitative multi-criteria analysis indicated that hydrated lime treatment provides a marginally greater benefit compared to the other two technologies at a slightly lower cost.

**Table 5:** Multicriteria table.

	Safety		Sanitation	Cost	Robustness	Deployment	Total
Criterion weight ^a,b^		5	5	4	4	3	
Ranking							
Lactic acid fermentation ^c^		5	5	2	3	2	76
Urea treatment ^c^		4	5	3	4	4	85
Lime treatment ^c^		4	5	4	5	3	90

^a^ These rankings were generated in consultation with emergency response personal [27]; ^b^ Criteria rank: (5: Critical, 4: Very Important, 3: Somehow important 2: Unimportant 1: Trivial); ^c^Alternatives rank: (5: Very good, 4: Good, 3:Ok, 2: Poor, 1: Very poor).

### 3.6. Study Limitations and Future Research

In this research, *E. coli* was used as the indicator organism for pathogens presence in faecal sludge on the basis of complying with the World Health Organisation guidelines for excreta [28]. However whilst *E. coli* is one of the traditional bacterial indicators, recent research has shown that there is limited correlation between *E. coli* absence and pathogen inactivation particularly for parasitic and viral pathogens. Additionally it has been suggested that bacterial indicators are poor indicators of the presence or absence of *Cryptosporidium* and *Giardia* [29]. This has implications for the conditions required to classify treated faecal sludge as “safe” from a public health perspective. The survival characteristics of bacterial, viral and parasitic pathogens vary in the environment indicating that no single microorganism can predict the presence of all pathogens. Whilst *E. coli* is not representative of viral and parasitic pathogens, it does correlate well with *Salmonella typhi*, *Salmonella paratyphi*, *Vibrio cholerae* and *Shigella* spp. which are also gram-negative bacteria and the primary cause of diarrheal infections throughout the world [30]. Further research using multiple and alternative indicators such as helminth eggs, *enterococci*, and bacteriophage (*Bacteroides fragilis*, coliphage and F-RNA phage) is recommended to determine the effectiveness of each of the proposed treatment methods to sanitize the sludge and reduce public health risk associated with different pathogens.

There were a number of limitations to the current study due to time and resource restrictions during the field trials. The fact that each experiment was undertaken by different co-workers in addition to the use of household and market sludge created some inconsistency between experimental design and laboratory analysis of the three treatment processes. Additionally, many of the analytical procedures used to characterize the sludge were developed for wastewater and hence incurred significant dilutions and associated errors when used to analyze faecal sludge. Whilst the current study achieved the aim of identifying possible faecal sludge treatment methods for applications in an emergency setting, further testing is recommended to optimize each process and devise reproducible operating procedures for full-scale emergency faecal sludge treatment processes. Additionally, as result of the effect of temperature on lactic acid and urea treatments, it is recommended that further testing be conducted at different temperatures to understand the relationship between sanitation time and temperature for these two treatments.

## 4. Conclusions

In the present study the sanitizing effect of lactic acid fermentation, urea and lime faecal sludge treatment methods were investigated. Field testing results indicated that lactic acid fermentation could reduce the *E. coli* count in faecal sludge to below the detection limit within 168 h at pH 4.2 and maintaining a lactic acid concentration of 47 g/L. The 2.5% *w*/*w* urea treatment provided >3.7 log removal for *E. coli* (CFU/mL) within 96 h with pH 9.4 conditions and ammonia concentrations between 4.5 and 5.8 g NH3-N/L. Total coliforms were reduced to below the detection limit during urea treatment after 168 h at pH 9.4 and ammonia concentration 7.7 g NH3-N/L. Hydrated lime treatment sanitised the faecal sludge to below detectable limits within 1 h for E. coli within at pH > 11 and within 2 h for total coliforms at pH > 11.5 conditions.

A qualitative multi-criteria analysis indicated hydrated lime treatment as the preferred emergency sanitation treatment due to its operational advantages such as short sanitation time, operational stability under temperature variation and aerobic conditions as well as low treatment cost. For longer-term (*e.g.*, refugee camp) sanitation systems however, urea or lactic acid treatment may be more suitable when the sanitised faecal sludge is to be used as a fertilizer. This research has demonstrated that all three sanitization methods have the potential to be implemented and be effective during an emergency situation and their respective suitability, will depend on the requirements for a particular emergency situation.

## References

[1] Fenner R.A., Guthrie P.M., Piano E. (2007). Process selection for sanitation systems and wastewater treatment in refugee camps during disaster-relief situations. Water Environ. J..

[2] Brown J., Jeandron A., Cavill S., Cumming O. (2012). Evidence Review and Research Priorities: Water, Sanitation and Hygiene for Emergency Response.

[3] Connolly M.A., Gayer M., Ryan M.J., Salama P., Spiegel P., Heymann D.L. (2004). Communicable diseases in complex emergencies: Impact and challenges. Lancet.

[4] Waring S.C., Brown B.J. (2005). The threat of communicable diseases following natural disasters: A public health response. Disaster Manag. Response.

[5] Vandenbergh P.A. (1993). Lactic acid bacteria, their metabolic products and interference with microbial growth. FEMS Microbiol. Rev..

[6] Alakomi H.L., Skyttä E., Saarela M., Mattila-Sandholm T., Latva-Kala K., Helander I.M. (2000). Lactic acid permeabilizes Gram-negative bacteria by disrupting the outer membrane. Appl. Environ. Microbiol..

[7] Zhu H., Hart C.A., Sales D., Roberts N.B. (2006). Bacterial killing in gastric juice—Effect of pH and pepsin on *Escherichia coli* and *Helicobacter pylori*. J. Med. Microbiol..

[8] Mante E.S., Sakyi-Dawson E., Amoa-Awua W.K. (2003). Antimicrobial interactions of microbial species involved in the fermentation of cassava dough into agbelima with particular reference to the inhibitory effect of lactic acid bacteria on enteric pathogens. Int. J. Food Microbiol..

[9] Ligocka A., Paluszak Z. (2005). Capability of lactic acid bacteria to inhibit pathogens in sewage sludge subjected to biotechnological processes. Bull. Vet. Inst. Pulawy.

[10] Soewondo P., Febriana A., Handajani M., Firdayati M., Bettendorf T.W.C., Otterpohl R. (2014). Faeces Treatment by lactic fermentation process and future perspectives of Terra Preta sanitation concept in Indonesia. Terra Preta Sanitation.

[11] Warren K.S. (1962). Ammonia toxicity and pH. Nature.

[12] Vinnerås B., Holmqvist A., Bagge E., Albihn A., Jönsson H. (2003). The potential for disinfection of separated faecal matter by urea and by peracetic acid for hygienic nutrient recycling. Bioresour. Technol..

[13] Zimmer M. (2000). Molecular mechanics evaluation of the proposed mechanisms for the degradation of urea by urease. J. Biomol. Struct. Dyn..

[14] Udert K.M., Larsen T.A., Biebow M., Gujer W. (2003). Urea hydrolysis and precipitation dynamics in a urine-collecting system. Water Res..

[15] Nordin A. (2010). Ammonia Sanitisation of Human Excreta: Treatment Technology for Production of Fertiliser. Ph.D. Thesis.

[16] Ottoson J., Nordin A., von Rosen D., Vinnerås B. (2008). Salmonella reduction in manure by the addition of urea and ammonia. Bioresour. Technol..

[17] Vinnerås B. (2007). Comparison of composting, storage and urea treatment for sanitising of faecal matter and manure. Bioresour. Technol..

[18] Bina B., Movahedian H., Kord I. (2004). The Effect of Lime Stabilization on the Microbiological Quality of Sewage Sludge. Iran. J. Environ.Health Sci. Eng..

[19] Fitzmorris K.B., Reimers R.S., Oleszkiewicz J.A., Little M.D. (2007). Decrease of time for pathogen inactivation in alkaline disinfection systems using pressure. Water Environ. Res..

[20] Mignotte-Cadiergues B., Maul A., Huyard A., Capizzi S., Schwartzbrod L. (2001). The effect of liming on the microbiological quality of urban sludge. Water Sci. Technol..

[21] Strande L., Roneteltap M., Brdjanovic D. (2014). Faecal Sludge Management: Systems Approach for Implementation and Operation.

[22] Sphere Project T. (2011). Excreta disposal. Humanitarian Charter and Minimum Standards in Humanitarian Response.

[23] Rand M.C., Greenberg A.E., Taras M.J. (2012). Standard Methods for the Examination of Water and Wastewater.

[24] Vinnerås B., Hedenkvist M., Nordin A., Wilhelmson A. (2009). Peepoo bag: Self-sanitising single use biodegradable toilet. Water Sci. Technol..

[25] Emerson K., Russo R.C., Lund R.E., Thurston R.V. (1975). Aqueous Ammonia Equilibrium Calculations: Effect of pH and Temperature. J. Fish. Res. Board Can..

[26] Sutton S. (2011). Accuracy of Plate Count. J. Valid. Technol..

[27] Anderson C., RedCross, IFRC (2013). Emergency Sanitation Project Meeting Minutes.

[28] WHO (2006). Guidelines for the Safe Use of Wastewater, Excreta and Greywater.

[29] Harwood V.J., Levine A.D., Scott T.M., Chivukula V., Lukasik J., Farrah S.R., Rose J.B. (2005). Validity of the indicator organism paradigm for pathogen reduction in reclaimed water and public health protection. Appl. Environ. Microbiol..

[30] Rodrigues V., Ramaiah N., Kakti S., Samant D. (2011). Long-term variations in abundance and distribution of sewage pollution indicator and human pathogenic bacteria along the central west coast of India. Ecol. Indic..

